# Study on Styrene-Butadiene-Styrene Modified Asphalt Binders Relaxation at Low Temperature

**DOI:** 10.3390/ma14112888

**Published:** 2021-05-27

**Authors:** Sylwia Dziadosz, Mieczysław Słowik, Filip Niwczyk, Marcin Bilski

**Affiliations:** 1 Faculty of Civil and Transport Engineering, Poznan University of Technology, Piotrowo 3, 60-965 Poznań, Poland; sylwia.k.dziadosz@doctorate.put.poznan.pl; 2 Institute of Civil Engineering, Faculty of Civil and Transport Engineering, Poznan University of Technology, Piotrowo 5, 61-138 Poznań, Poland; mieczyslaw.slowik@put.poznan.pl; 3 STRABAG Ltd., Prymasa Wyszyńskiego 1A, 62-420 Strzałkowo, Poland; filip.niwczyk@strabag.com

**Keywords:** force-ductility-relaxation test, styrene-butadiene-styrene modified bitumen, rolling thin film oven test, generalized Maxwell model, rheological properties at low temperature

## Abstract

The paper presents the results of laboratory investigation on asphalt binders relaxation at low temperature, carried out in a ductilometer using the tensile test with continuous force measurement. Polymer modified asphalt binder samples consisting of a 50/70 penetration grade bitumen mixed with a concentrate of styrene-butadiene-styrene (SBS) modified bitumen—a 160/220 penetration grade bitumen modified with a SBS copolymer in the amount of 9%—were tested. Therefore, polymer modified binders containing 3%, 4.5%, 6% and 7.5% SBS, respectively, were obtained and investigated. Tensile tests were performed at −16 °C on samples before aging and subjected to short-term aging (RTFOT). Test results in the form of relaxation curves have been mathematically described using a modified generalized Maxwell model. Based on the acquired results, it was shown that the increase of the SBS copolymer content in asphalt binder precipitates the relaxation process, while aging slows down this phenomenon. It has also been proven that with increased content of SBS elastomer in asphalt binder, the effect of short-term aging on binder’s stress relaxation ability at low temperatures is reduced.

## 1. Introduction

One of the leading factors severely damaging the asphalt and concrete road pavements is the impact of heavy traffic and high temperature gradient. The influence of these stimuli constitutes the reason why a road surface breaks and deforms permanently [1,2].

The bituminous binder is considered the most significant part of the asphalt mixture in terms of durability and crack resistance against damages occurring due to fatigue or as a result of low temperature [3,4]. As a consequence of the increasing traffic each year, there is a demand for alternative methods of changing the properties of asphalt binders through various modifiers like polymers or natural asphalt [4,5,6]. These modifications can affect reduction of the deterioration of the asphalt mixture during its use, which in turn reduces the economic cost of maintaining the pavements.

In recent years, research works have focused on determining the exact impact of modifiers on the rheological properties of bituminous binders. Yilmaz and Celoglu [4] examined the effect of additions of assorted natural asphalt and polymers on the durability improvement of the binder, while Słowik and Bilski [7] analyzed how Gilsonite and Trinidad Epuré natural asphalts positively affect its properties after short-term and long-term aging. Moreno-Navarro, Sol-Sanchez and Rubio-Gamez [8] in their research focused on the benefits of using polymer modified binders in the context of long-term performance of pavements. Their study concluded that the effectiveness of bitumen modification with polymers depends on climatic conditions. Toraldo and Mariani conducted laboratory investigation in which they focused on the influence of ethylene-vinyl acetate and Low-density polyethylene polymers on improving the properties of asphalt binders at in-service temperatures [6]. Ranieri and Celauro have confirmed that polymer additives improve the mechanical performance of asphalt mixtures, even if during their production an average quality aggregate and a binder with a high penetration grade was used [5]. Iwański et al. [9], as well as Mazurek and Iwański [10], determined viscoelastic parameters of asphalt binders with different synthetic waxes and looked for the best wax additive to foam bitumen. For example, the Fischer–Tropsch synthetic wax content of 2.5% induced an improvement in bitumen ability to foam.

Currently, the modifiers market is dominated by the styrene–butadiene–styrene copolymer (SBS) [11,12,13,14,15,16,17]. With the use of SBS for modification, an asphalt binder with improved rheological properties is obtained. These improved properties include viscosity, adhesion to aggregates and cohesion. As a consequence, the SBS modified asphalt mixture is characterized by increased resistance to permanent deformation and thermal fracturing as well as fatigue durability [1,3,8,11,13]. The SBS copolymer modifies the binder structure. It expands and absorbs maltenes from the heated binder, increasing its volume. At a content of 6% of the SBS, a copolymer is at the dispersing phase and forms a continuous web in the asphalt binder, which has a significant impact on its properties [14]. The extensive research of rheological properties of SBS modified binders was performed by Airey [15]. He observed that aging of the material results in a reduction of the molecular size of the SBS copolymer with a decrease in the elastic properties of asphalt binder. Shan et al. [16] showed that with an increase in the SBS copolymer content, the nonlinear viscoelasticity of asphalt binder increases. Yan et al. described the effect of the modifier such as a composite of SBS and other polymers such as polypropene, terpolymer or oxidized polyethylene wax on the asphalt binder cracking [18]. The laboratory investigation proved that these additives can reduce the rate of damage accumulation in asphalt mixture and improve the resistance to cracking.

Furthermore, scientists analyzed the influence of modification on properties of bitumen in low temperatures. Marasteanu, Falchetto, Balamurugan and Negulescu studied the impact of the cooling medium (ethanol, potassium acetate and air) and size of a sample on the durability of asphalt binder at low temperature with modified Bending Beam Rheometer (BBR) and Direct Tension Tester (DTT) [19]. The major conclusion drawn from their research was that while the results of tests of the binders modified with air and potassium acetate are similar, whereas the results of the ethanol probe are different. This particular alcohol has detrimental effects on binder strength due to diffusion. Lu, Uhlback and Soenen investigated bitumens with wax addition at low temperatures by comparing the results of two tests. They established a correlation between complex modulus measured by Dynamic Shear Rheometer (DSR) with 4 mm parallel plates and creep stiffness acquired from BBR test [20]. Laukkanen et al. examined the rheological properties of SBS modified asphalt binders at low temperatures using a DSR apparatus and confirmed that the low temperature stiffness is significantly reduced through the addition of this elastomer [11]. Nian et al. analyzed the impact of freeze–thaw cycles on durability of SBS modified binders and proved that SBS elastomer can expand the elasticity and decrease the bitumen’s temperature susceptibility [1]. Nevertheless, the subject of low temperature properties of modified bitumen is infrequently studied by the scientists [9]. In the study [21], the authors sought to evaluate the relationship between Δ*T*c and the Glover-Rowe parameter, which have some correlation with cracking, reinforcing the importance of considering time and temperature. On the other hand, the authors of study [22] analyzed the influence of aging process with particular focus on the influence of UV radiation on asphalt binders with different modifiers. In another case, the authors of studies [23,24] successfully used Dynamic Shear Rheometer (DSR) to determine bitumen relaxation.

There is a possibility of modeling phenomena occurring during loading and unloading of the samples of asphalt binders and mastics using rheological models. Due to viscoelastic properties of these materials, one can find quite a few examples of using different rheological models in the literature, such as Maxwell, Kelvin-Voigt or Burgers models, for mathematical description of their behavior. Scientists modify them in order to achieve the most precise descriptions of processes (e.g., relaxation, creep, recovery) occurring in asphalt binders. Lagos-Varas et al. have developed a modified Burgers rheological model for asphalt mastics, which allows for optimization of their composition in regard to their performance and mechanical properties for asphalt pavements [25]. Qinglong, Jinglian and Xin have chosen the Burgers model for modeling of creep and relaxation observed at high temperatures concerning asphalt binders modified with the SBS copolymer and mineral fillers, whereas the Maxwell model has been chosen as the more effective one for unmodified mastics [26]. Therefore, variation of models (their combination and number of basic single parameter elements) is dependent on the specificity and composition of the tested material. The Maxwell model, which is established based on the combination of springs and dashpots, reflects the most adequate representation of the relaxation curves [14,27]. Bans, Kenz and Hu [28] have proven that the Maxwell model predicts that stress decays exponentially with time, which is accurate for many materials, especially polymers (polymer modified bitumen).

The principal objective of the this paper is to enhance knowledge about the aforementioned issue. The innovation of the paper is the original (non-standard) methodology of research consisting in direct observation of the relaxation phenomenon on asphalt binder samples subjected to straight stretching in a ductilometer at low temperature (−16 °C). Furthermore, the impact of high copolymer content in samples on asphalt binders properties was investigated. The tested asphalt binders were not modified directly with the addition of SBS copolymer, but were combined (blended) with highly modified bitumen (concentrate) with a specified content of SBS copolymer (9.0 ± 0.2%) produced in a refinery (process of SBS modification was conducted by industrial method). The primary laboratory apparatus used in the research was a ductilometer. The comparative studies also took into consideration the influence of short-term aging occurring during technological processes, simulated using the RTFOT (rolling thin film oven test) laboratory method on the relaxation of the binder. The influence of short-term aging process on the changes in the properties of the asphalt binders containing the SBS additive was investigated in the paper. Results of the observations on two levels of aging asphalt binders were deemed interesting and were, therefore investigated in the article. Study of the impact of long-term aging process simulated in laboratory using the Pressure Ageing Vessel (PAV) method is planned for further stages of the research. Test results, shown as relaxation curves, have been mathematically described using a generalized Maxwell model, achieved by parallel connection of two, three or four Maxwell elements. Using this method, models with four, six and eight parameters have been prepared. Further considerations focused on modification of the four-parameter generalized Maxwell model. During the first attempt, time domain has been changed from *t*^1^ to *t^β^*, where the *β* exponent is the fifth parameter of the analyzed model. Since the values of parameter *β* achieved at the end of modeling were within the range of 0.47 to 0.54, during the second attempt the model has been simplified and four parameters were used with a defined value of time exponent *β* = 0.5 (achieving time domain *t*^0.5^). Using modified equations in the four-parameter model allowed to achieve a better fit for relaxation curves prepared by means of empirical method than in the case of classic generalized Maxwell model with six parameters.

## 2. Materials and Methods

### 2.1. Materials

In the research program, the following asphalt binders were chosen for the examination: 50/70 penetration grade bitumen (Lotos, Gdańsk, Poland), highly modified asphalt binder (Lotos, Gdańsk, Poland) containing 160/220 penetration grade bitumen and 9% of styrene-butadiene-styrene copolymer with linear structure (hence labeled in the present paper as a “concentrate”). The first step taken to prepare the polymer modified bitumen specimens was heating them up. The 50/70 penetration grade bitumen was heated up to 140 °C, and a concentrate was heated up to 190 °C until they became fully liquid. Secondly, both materials were put together in previously prepared aluminum containers in various weight proportions in order to obtain polymer modified bitumens containing the following amount of SBS copolymer: 3%, 4.5%, 6% and 7.5% of the mixture weight (respectively 33.3%, 50%, 66.7% and 83.3% of concentrate mass content in modified binder). Then, a specialized laboratory agitator was used to blend both materials until homogenous mixtures were obtained. After finishing the blending process, each mixture was covered with an aluminum lid and stored in a dark locker at room temperature to retain its obtained properties.

### 2.2. Experimental Methods

#### 2.2.1. Penetration and Softening Point Tests

Penetration and ring and ball softening point tests were conducted to classify previously prepared asphalt binders. Penetration of every examined binder was determined at the temperature of 25.0 ± 0.1 °C according to EN 1426:2015 [29] making seven tests for each sample. Ring and ball softening point tests were conducted using the method described in EN 1427:2015 [30] using four specimens.

#### 2.2.2. RTFOT-Short-Term Aging Simulation

For every previously prepared asphalt binder specimen, simulation of aging in laboratory condition was performed using the Rolling Thin Film Oven Test (RTFOT). The test was conducted using procedure described in EN 12607-1:2014 [31]. For the detailed RTFOT procedure unaged bituminous binder samples in cylindrical glass vessels were taken, and these vessels were placed in a rotating carriage inside an oven. The carriage rotated the samples within the oven at (163 ± 1) °C for 75 min. The samples were then poured into previously prepared aluminum containers and stored for further tests.

#### 2.2.3. The Relaxation Test Using Ductilometer

**Figure 1:**
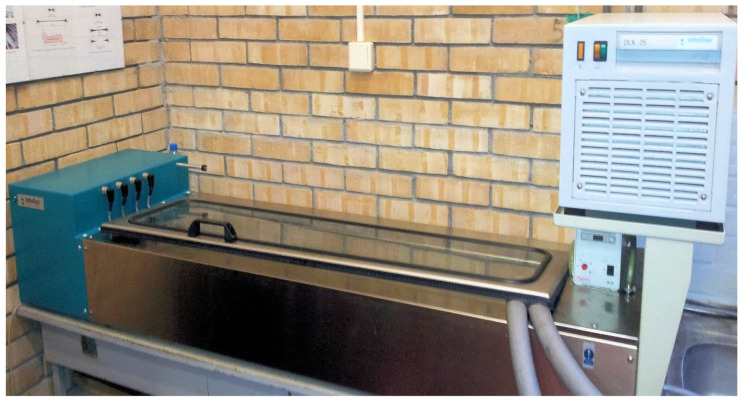
Ductilometer with an auxiliary cooling unit.

**Figure 2:**
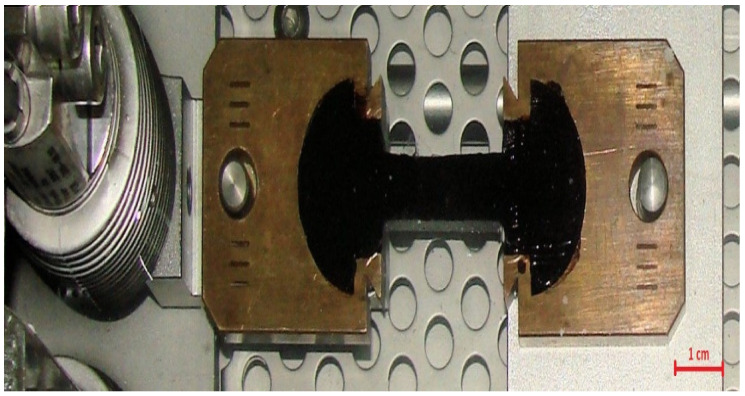
Asphalt binder sample during the ductilometer test.

In order to examine relaxation at −16 °C of previously prepared asphalt binders, forms and laboratory devices (Infratest, Brackenheim, Germany) in accordance with EN 13589:2008 [32] were used. The ductilometer presented in Figure 1 has been modified by adding a second cooling unit, therefore allowing it to achieve the temperature of −16 °C. The temperature for the study was set at −16 °C so that later, in the next stage of the authors’ research, the results could be correlated with the values obtained by using the BBR rheometer (Cannon Instrument Company, State College, PA, USA). Applied test methodology was developed on the basis of the Direct Tension Test described in American Association of State Highway and Transportation Officials (AASHTO) T 314 [33]. Before the tests were carried out, ductilometer was connected to an additional cooling system to enhance its capabilities of lowering the temperature. It was then filled with a liquid consisting of 70% distilled water and 30% ethylene glycol. Firstly, the ductilometer and cooling system were turned on and the temperature of −16.0 °C was set. After cooling the liquid, the temperature fell down to −16.0 ± 0.5 °C, and binder samples, previously prepared in brass forms, were put into the ductilometer bath. Then, the samples were stored for thirty minutes. During that time, the computer and software dedicated to carrying out the test in the ductilometer were turned on. After successful calibration of the ductilometer measuring sensors, the asphalt binder samples prepared in brass forms were put onto test places and two lateral elements of the forms were gently removed. Next, the ductilometer cover was closed and the test was carried out. Each sample was extended individually with a speed of 1 mm/min until the tensile force of 50 N was obtained. An example of a test sample can be seen in Figure 2. The tensile speed and the value of the final tensile force were selected experimentally to avoid cracking of the samples and to achieve strain value not exceeding 0.1% (low deformation) in order to calculate values of the tensile stress. When tensile force reached a value of 50 N, the constant elongation value of the specimen was retained for at least 20 min and during that period the force values were recorded. Thus, this original measurement method is called a force-ductility-relaxation test. Afterwards, the results obtained from the computer program were taken for further analysis, and the whole procedure starting from samples being thermostated was run over again for the next binder specimen. The examinations were finished when four unaged samples and four samples subjected to aging of each binder were tested.

#### 2.2.4. Mathematical Description of Relaxation Curves

**Figure 3:**
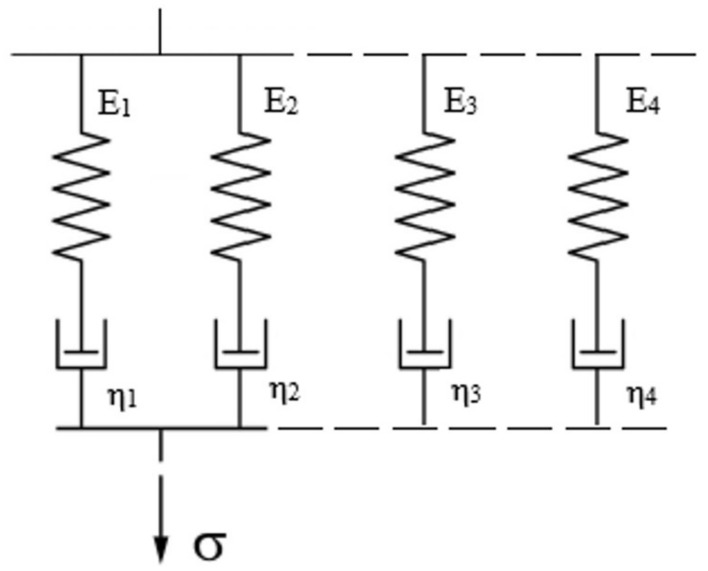
Combination of elastic and viscous elements in generalized Maxwell models with 4, 6 and 8 parameters [14].

When describing relaxation phenomenon, generalized Maxwell models with 4, 6 and 8 parameters were used. The method of combining elastic (Hookean solid) elements (characterized by modulus of elasticity *E_i_*) as well as viscous (Newtonian liquid) ones (characterized by dynamic viscosity *η_i_*) is presented in Figure 3.

Presented models do not show a permanent viscous or elastic bonding. They describe all phenomena characteristic for viscoelastic bodies: elastic strain, immediate and delayed recovery, creep, permanent strain and complete relaxation. Generalized Maxwell models combined in a parallel manner, are best suited for relaxation, when the value of strain is constant, and stress is a time variable.

Complete (tensile) stress in the model at *t* moment is the sum of stresses transferred by *n* Maxwell elements and is described using Equation (1) or Equation (2) [34]:

(1) $$\sigma \left(t\right)=\varepsilon_{0}\overset{n}{\underset{i=1}{\sum }}E_{i}exp\left(-\frac{E_{i}}{\eta_{i}}t\right)$$

(2) $$\sigma \left(t\right)=\varepsilon_{0}\overset{n}{\underset{i=1}{\sum }}E_{i}exp\left(-\frac{t}{\tau_{i}}\right)$$

where:

1. *σ*(*t*)—stress at *t* moment;
2. *ε*_0_—maximum strain obtained as a result of stretching the samples (strain with a constant value for 20 min to observe relaxation);
3. *t*-time;
4. *E_i_*—modulus of elasticity of Hooke’s element *i*;
5. *η_i_*—dynamic viscosity of Newton’s element *i*;
6. $\tau_{i}=\frac{\eta_{i}}{E_{i}}$ is the time of relaxation of Maxwell element *i*.

The values of parameters in generalized Maxwell models were calculated using iteration method, which led to minimization of sum of the squared deviations (SSq) and using the Nonlinear Least Squares Regression online software (Nonlinear Least Squares Regression (Curve Fitter), https://statpages.info/nonlin.html).

## 3. Results and Discussion

### 3.1. Penetration and Softening Point

**Figure 4:**
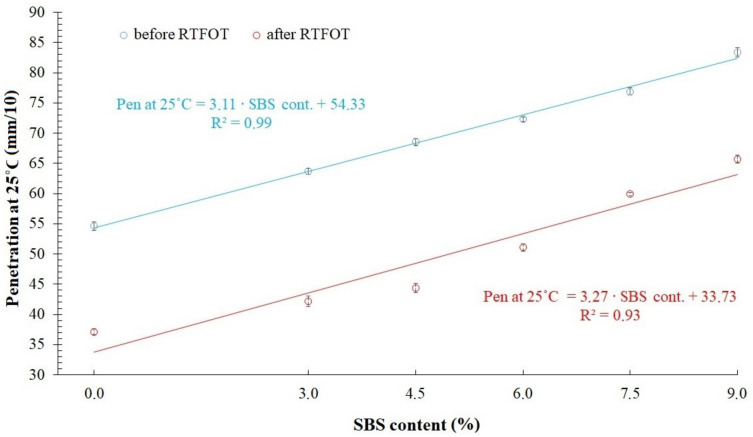
Penetration values of tested asphalt binders.

**Figure 5:**
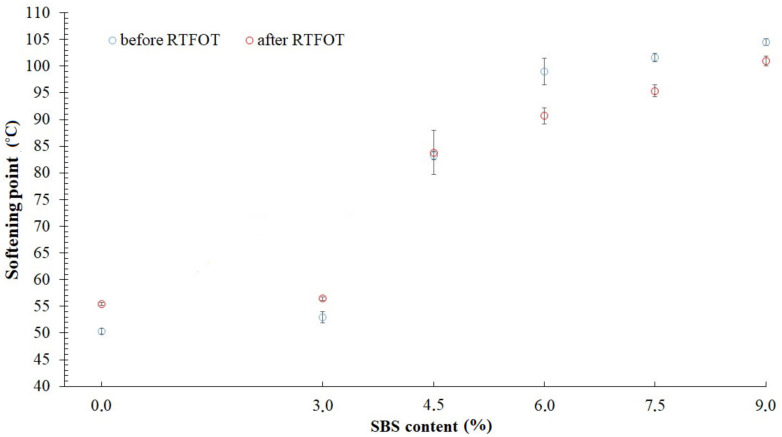
Softening point values of tested asphalt binders.

Obtained values of penetration are shown in Figure 4 and Table 1. Softening point temperatures are demonstrated in Figure 5 and presented in Table 1. Statistical analysis was prepared for each value, which consists of discarding results with gross error and determining expanded uncertainty using t-Student’s distribution quantile. Gross error has been specified using Grubbs’ test. Assumed relevance level amounted to *α* = 0.05. Every tested bitumen, unaged and after RTFOT simulation, fulfilled requirements included in specification EN 12591:2009 [35].

After the simulation in the RTFOT device, the penetration decreased due to the stiffening of the asphalt binder. The softening point temperature increased after short-term aging. However, along with the enhancement of the elastomer content in the tested samples, values began declining. In highly modified bitumens (with 6%, 7.5% SBS and 9% concentrate) a continuous polymer phase occurs, which can explain lower differences in the increase of Softening Point T_R&B_ value.

### 3.2. Relaxation Test Results

**Figure 6:**
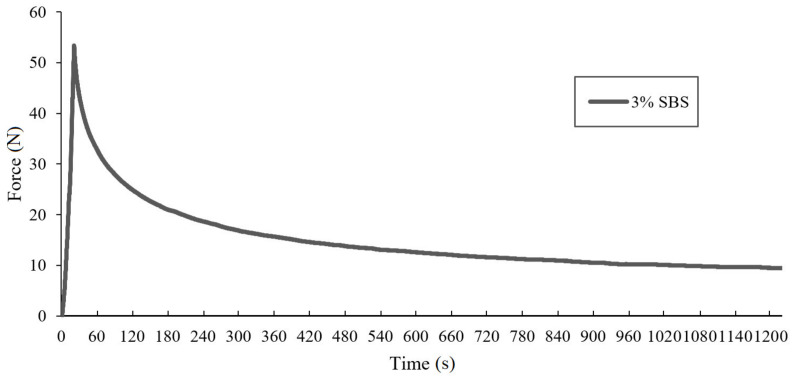
Results obtained from the force-ductility-relaxation test at −16 °C conducted for one sample of the asphalt binder modified with 3% of SBS copolymer.

For every analyzed binder four unaged samples and four samples subjected to RTFOT aging simulation were tested. The average values of force for each binder were calculated using attained results for every particular probe. The full test results for one selected sample (containing 3% of SBS copolymer) was presented in Figure 6.

**Figure 7:**
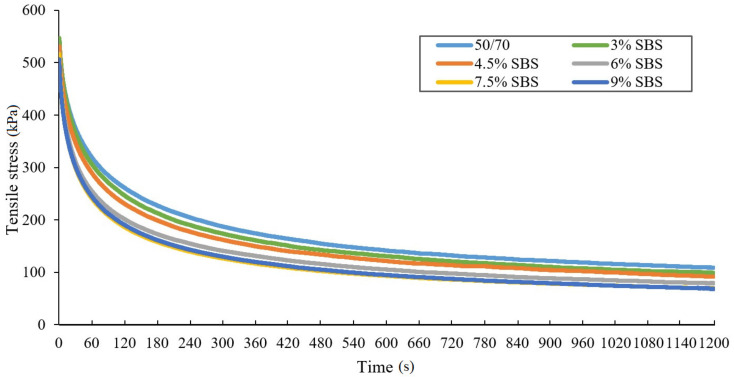
Relaxation curves of unaged bituminous binders obtained from the force-ductility-relaxation test conducted at −16 °C.

**Figure 8:**
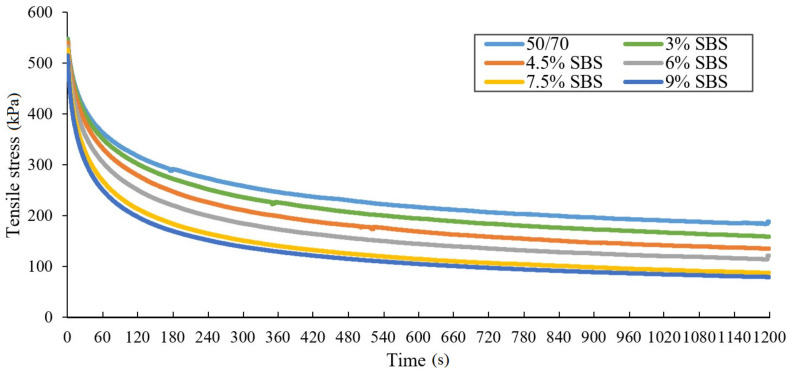
Relaxation curves of RTFOT aged bituminous binders obtained from the force-ductility-relaxation test conducted at −16 °C.

Due to ductilometer mechanisms’ inertia force, values obtained from the test at the start are higher than 50 N, but the influence of this phenomenon on final stress results can be considered as insignificant. The maximum difference of averaged tensile force in the analyzed binders at the end of the stretching process amounted to 4.5 N. The obtained results enabled drawing the relaxation curves, at the time of observation equal to 1200 s (Figure 7 and Figure 8).

Tensile stress was calculated according to Equation (3).

(3) $$\sigma =\frac{F}{A}$$

where *σ*—tensile stress [Pa], *F*—tensile force [N] and *A*—cross-sectional area of the sample [m^2^]. Brass forms that were used guaranteed that cross-sectional area of every tested sample was 10^−4^ m^2^. Due to the small deformation of the samples, the assumption concerning the invariant cross-section was adopted [36,37].

Analysis of the relaxation curves of asphalt binders tested at −16 °C has shown that tensile stress values are declining rapidly immediately after the force of 50 N is reached (tensile stress value decreases by a half before the first three minutes pass from the beginning of recording the force needed to retain the sample’s constant elongation). In addition, one can observe that the higher SBS copolymer content, the faster the stress relaxation occurs. This phenomenon proves that the SBS modification of the binder had a positive effect not only on the temperature susceptibility, but also on the acceleration of the relaxation phenomenon. The tensile stress value difference between the 50/70 penetration grade bitumen and the asphalt binder containing 9% of SBS during first 60 s amounts to 120 kPa before aging and 180 kPa after RTFOT aging. Application of a SBS modifier visibly improves asphalt’s ability to relax faster in low temperature. This is a desirable occurrence, as it prevents accumulating stress in asphalt layers of the pavement, exceeding its low temperature crack resistance.

When analyzing the differences between individual stress curves before aging, it has been noted that the variation between the relaxation curve for the concentrate and other samples decreases with higher SBS content. For 50/70 penetration grade bitumen, the difference was 38%; however, for samples containing 7.5% of SBS copolymer, it was only 3%-curves overlap. This occurrence is associated with an alteration in the binder structure after exceeding the modification limit with the 6% of SBS copolymer—the polymer becomes a dispersing phase. Increasing the content of the SBS copolymer in the binder above by about 7.5% has a nonsignificant influence on the relaxation phenomenon.

Furthermore, when analyzing the curves in the context of the impact of RTFOT aging simulation, it has been found out that the lower the amount of SBS copolymer in the asphalt binder, the higher the difference in average tensile stress values between unaged samples and those subjected to short-term aging. It varies from 14% for the concentrate to 42% for 50/70 penetration grade bitumen. Moreover, average tensile stress values determined after 20 min of maintaining constant elongation for probes after RTFOT aging are higher than for unaged specimens. The difference between unaged samples and those subjected to short-term aging decreases with the growth of SBS copolymer content from 7.9 N for 50/70 to 1.1 N for concentrate (containing 9% of SBS).

The study shows that with the growth of styrene–butadiene–styrene copolymer percentage content in the mixture, weight disparities between aged and unaged samples are decreasing. Consequently, this modification reduces the negative effect of short-term aging on the relaxation phenomenon and reduces the increase in the value of tensile stress. In summary, highly modified asphalt binders (6% and 7.5% of SBS content), taking into consideration RTFOT short-term aging process, are characterized by better performance than classic SBS modified binders (3%, 4.5% of SBS content). They are also characterized by higher degradation resistance under the effect of the aging process. Therefore, it is possible to expect that asphalt pavements using highly modified binders will have a higher durability.

### 3.3. Relaxation Curves Mathematical Description

Values of parameters of a generalized Maxwell model (in versions with four, six and eight parameters) calculated with Equation (1) and values of statistical parameters characterizing fitting of modelled relaxation curves to the ones achieved by using their own empirical method (R^2^ coefficient of determination, root mean square error–RMS Error and sum of the squared deviations–SSq) for exemplary, unaged asphalt binder with a 4.5% SBS copolymer are summarized in Table 2, Table 3 and Table 4.

In line with the expectations, the best fitting of modelled relaxation curve with test results was obtained by using a generalized Maxwell model with eight parameters. This was confirmed by values of statistical parameters: the highest value of R^2^ coefficient of determination and lowest values of RMS Error and SSq sum of squares. Despite the fact that in each of the used models, high values of coefficient of determination were observed (in all cases R^2^ > 0.99), the differences between them are significant which is demonstrated by two other parameters (e.g., there is a 16 times difference in RMS Error values and 160 times difference in SSq values). It should be kept in mind that increasing the number of parameters in a model results in achieving a better fitting of curves obtained via the analytical and empirical methods. At the same time a large quantity of parameters means that arrangement of their values shows a visible diversification. That is why, an attempt was made to modify the Equation (1) by changing the domain of time from *t*^1^ to *t^β^*. For *n* = 2 Equation (1) will take the following form:

(4) $$\sigma \left(t\right)=\varepsilon_{0}\left[E_{1}exp\left(-\frac{E_{1}}{\eta_{1}}t^{\beta }\right)+E_{2}exp\left(-\frac{E_{2}}{\eta_{2}}t^{\beta }\right)\right]$$

Table 5 matches values of parameters used in model described by Equation (4) with R^2^, RMS Error and SSq statistical values for exemplary, unaged asphalt binder containing a 4.5% of SBS copolymer.

It can be concluded that a model with five parameters described by Equation (4) allows to achieve better fitting of curves obtained via the analytical and empirical methods than in the case of a generalized Maxwell model with six parameters described by Equation (1) and a slightly worse fitting than in the case of a model with eight parameters, which can be seen when comparing relevant RMS Error and SSq values.

**Figure 9:**
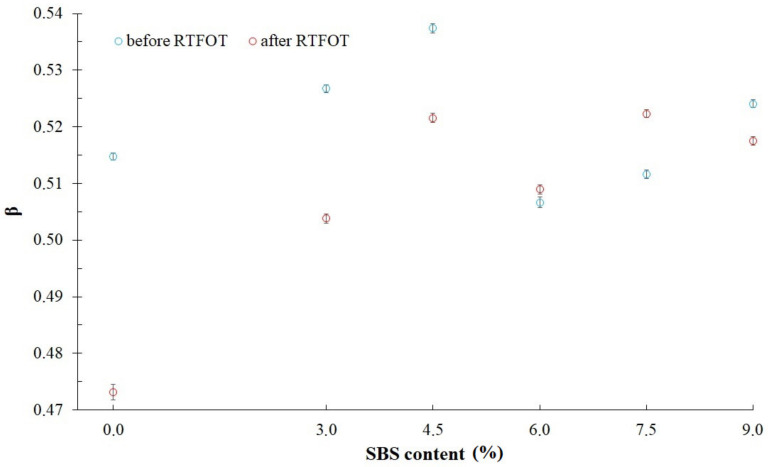
Values of time exponent (parameter *β*) in a modified generalized Maxwell model with five parameters.

Figure 9 and Table 6 present values of *β* parameter, which is the time exponent, used in a five-parameter model described with Equation (4). It has been found that values of parameter *β* set for all analyzed asphalt binders are similar and are in the range of 0.47 to 0.54. Equally important is the fact that the correlations between parameter *β* and SBS copolymer content in the asphalt binder or its aging have not been observed. That is why in the next step, it has been decided to use a constant value of *β* parameter (*β* = 0.5), obtaining a modified generalized Maxwell model with four parameters, described by the following equation:

(5) $$\sigma \left(t\right)=\varepsilon_{0}\left[E_{1}exp\left(-\frac{E_{1}}{\eta_{1}}\sqrt{t}\right)+E_{2}exp\left(-\frac{E_{2}}{\eta_{2}}\sqrt{t}\right)\right]$$

Table 7 matches values of parameters calculated by using the model described by Equation (5) with R^2^, RMS Error and SSq statistical values for exemplary, unaged asphalt binder with a 4.5% SBS copolymer.

**Figure 10:**
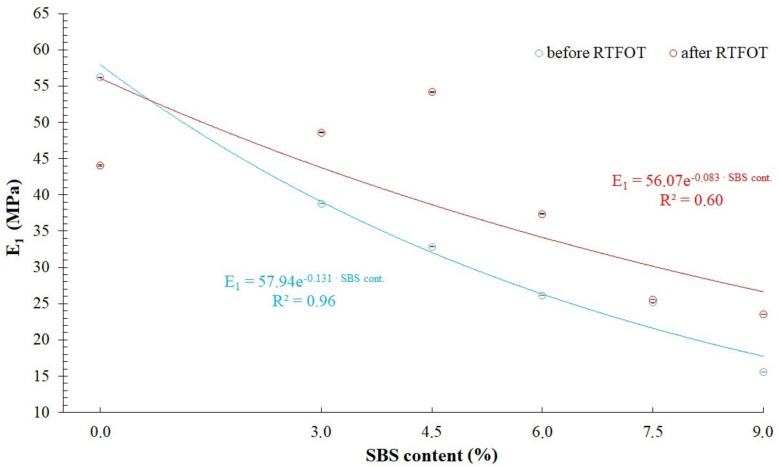
Values of modulus of elasticity (parameter *E*_1_) in a modified generalized Maxwell model with four parameters.

**Figure 11:**
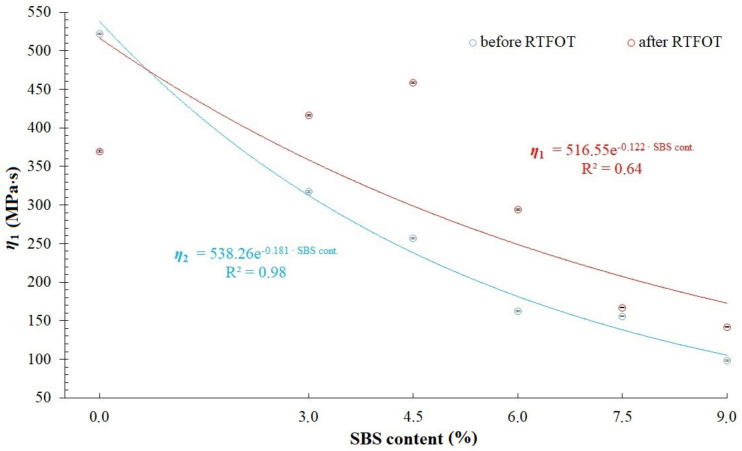
Values of dynamic viscosity (parameter *η*_1_) in a modified generalized Maxwell model with four parameters.

**Figure 12:**
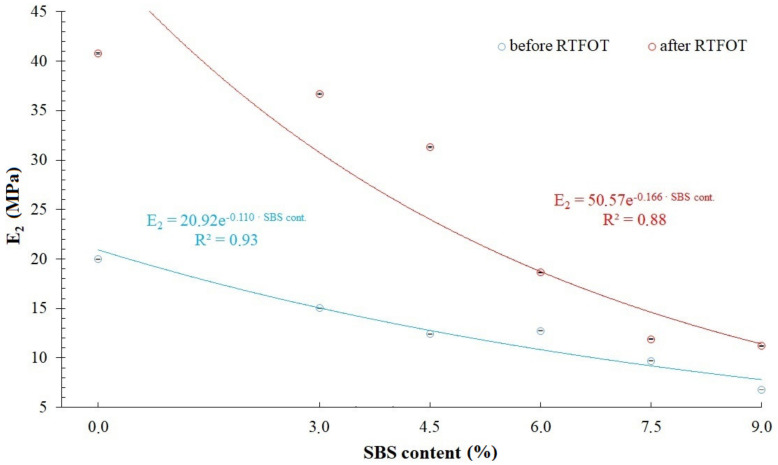
Values of modulus of elasticity (parameter *E*_2_) in a modified generalized Maxwell model with four parameters.

**Figure 13:**
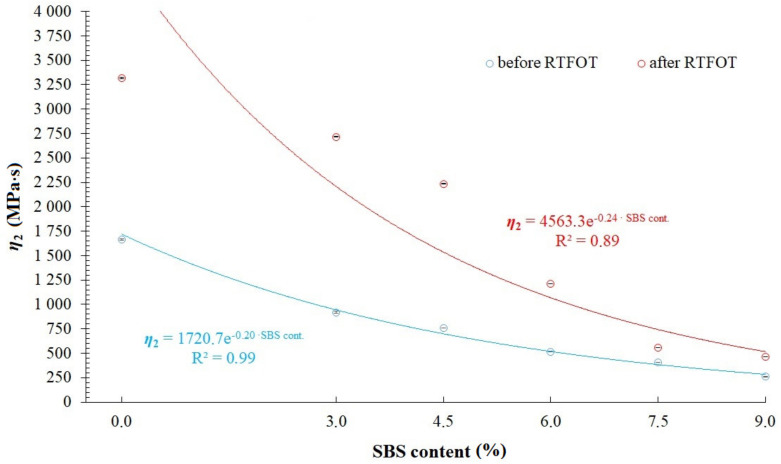
Values of dynamic viscosity (parameter *η*_2_) in a modified generalized Maxwell model with four parameters.

When comparing RMS, SSq and R^2^ values available in Table 2, Table 3, Table 4 and Table 7, it has been found that the modified generalized Maxwell model with four parameters, which was described by Equation (5), allows for acquisition of a very good fitting of relaxation curves obtained via the analytical and empirical methods, conceding slightly to the model with five parameters described by Equation (4) and generalized Maxwell model with eight parameters. That is why, further in this study, analysis has been performed on values of *E*_1_, *η*_1_, *E*_2_, *η*_2_ (assuming that *E*_1_ > *E*_2_) of a modified generalized Maxwell model described by Equation (5). These values are presented in Figure 10, Figure 11, Figure 12 and Figure 13 and Table 8, respectively.

Analysis of values of parameters in the modified generalized Maxwell model described by Equation (5) demonstrated in Figure 10, Figure 11, Figure 12 and Figure 13, shows that they are dependent on the content of SBS copolymer in modified bitumen. Furthermore, the short-term aging process (RTFOT) used on the studied binders also has an effect on the their values. In case of parameters *E*_1_ and *η*_1_ (Figure 10 and Figure 11), simple correlations have been observed only in the case of unaged asphalt binders. It has been found that increasing the content of SBS copolymer in modified bitumen lowers values of parameters *E*_1_ and *η*_1_. In case of binders subjected to the RTFOT aging process, correlations between parameters *E*_1_ and *η*_1_ have a different character. When increasing the content of SBS copolymer in modified asphalt binder in the 0 to 4.5% range, an increase of *E*_1_ and *η*_1_ values has been observed. Further, increase of the SBS copolymer content in modified binder (in the 4.5 to 9% range) results in a significant decrease of *E*_1_ and *η*_1_ values. The effect of the aging process simulated by RTFOT method on *E*_1_ and *η*_1_ values is also interesting. In the case of 50/70 penetration grade bitumen, aging results in a decrease of values of both parameters. On the other hand, in the case of all analyzed binders containing SBS copolymer, an increase of values of these parameters has been observed. In case of parameters *E*_2_ and *η*_2_ (Figure 12 and Figure 13), simple correlations have been observed both in the case of unaged asphalt binders and those aged using the RTFOT method. It has been found that increasing the content of SBS copolymer in modified asphalt binder lowers values of parameters *E*_2_ and *η*_2_. On the other hand, as a result of aging using the RTFOT method, an increase of values of E_2_ and *η*_2_ parameters in all analyzed binders has been observed. The above-described observations show that one of the elements of the Maxwell model (in which an elasticity element with a higher value of modulus of elasticity *E*_1_ is present) exhibits a higher sensitivity to changes caused by aging of binders than the latter one (in which an elasticity element with a lower value of modulus of elasticity *E*_2_ is present).

**Figure 14:**
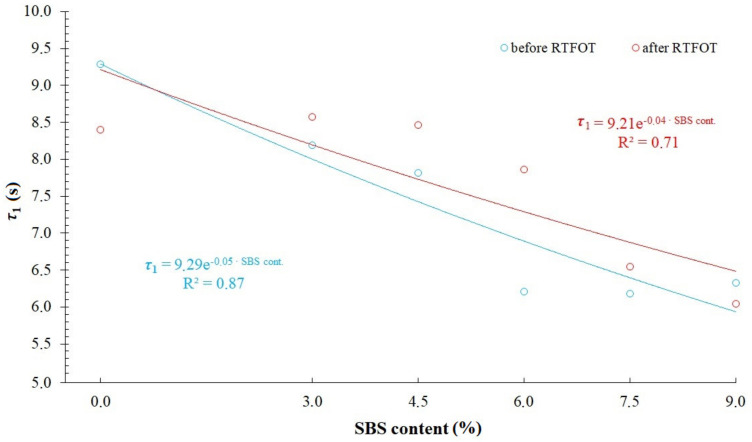
Relaxation time *τ*_1_ = *η*_1_/*E*_1_ in modified generalized Maxwell model with four parameters.

**Figure 15:**
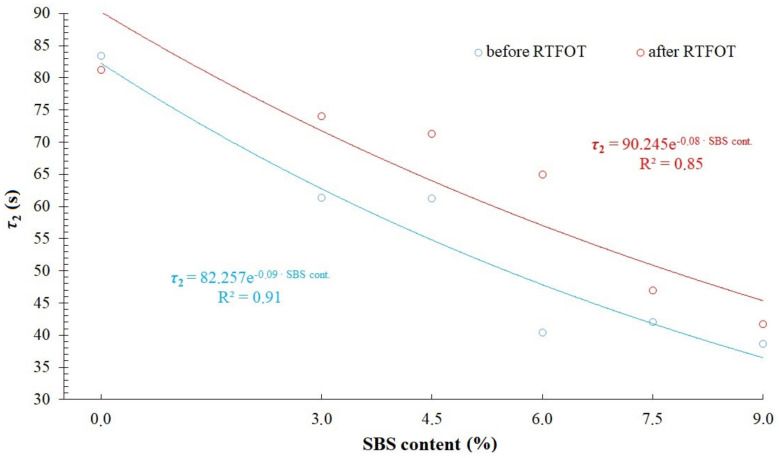
Relaxation time *τ*_2_ = *η*_2_/*E*_2_ in modified generalized Maxwell model with four parameters.

Figure 14 and Figure 15 and Table 9 show values of relaxation time, *τ*_1_ and *τ*_2_, respectively, calculated on the basis of modelling results compared in Figure 10, Figure 11, Figure 12 and Figure 13. A general trend has been observed by which values of relaxation time decrease with an increase of SBS copolymer content in the asphalt binder. Decrease of relaxation time should be considered as a positive phenomenon, allowing for a faster reduction of tensile stress in asphalt binder (e.g., arising from a sudden temperature drop of the asphalt pavement). It should be remembered that asphalt binder properties are primarily responsible for asphalt pavement resistance against low temperature cracking. Influence of the aging process in case of 50/70 penetration grade bitumen and modified binders on relaxation time values is varied. Shortening of relaxation times in case of 50/70 penetration grade bitumen and their increase in case of analyzed binders containing SBS copolymer has been observed. This is caused by a different structure of 50/70 bitumen and modified binders. In the case of modified bitumen, different degrees of polymer saturation in asphalt binder can be differentiated. In highly modified bitumens, a continuous polymer phase occurs, that is why the binder has a more homogenous structure. Aging process has an effect on both the change of base bitumen properties as well as degradation of copolymer. These effects, however, can be varied, depending on the content of SBS copolymer in the modified asphalt binder. This can explain the differences observed in Figure 14 and Figure 15.

## 4. Conclusions

The purpose of this research work was to assess the low temperature properties of SBS modified asphalt binders and their capability to relaxation. Based on the laboratory tests that were carried out, analysis of the obtained results of laboratory tests and mathematical description of experimental relaxation curves, the following conclusions can be drawn:

1. The larger the SBS copolymer content in the binder the faster the relaxation phenomenon occurs;
2. Simultaneously with the change in the structure of the binder with 7.5 and more percentage of SBS copolymer in modified binder weight, differences in relaxation curves become negligible regardless of the polymer content in the sample (7.5% or 9% in this research)-increasing the SBS content above this limit will not affect the relaxation phenomenon;
3. Aging simulated by Rolling Thin Film Oven Test method causes the relaxation phenomenon to slow down;
4. Relaxation of the asphalt binder occurs the fastest immediately after the stop of the elongation process, then it slows down. In none of the tested binders, the tensile stress decreased to zero in established measurement conditions (time of relaxation of 1200 s);
5. With the increase in the content of the SBS copolymer, the influence of the short-term aging on the final value of the recorded force in the test conducted at −16 °C has been reduced;
6. SBS copolymer reduces the susceptibility of binders to altering their rheological properties occurring under the influence of aging—the divergences between relaxation curves of unaged samples and those subjected to RTFOT aging simulation decrease with a higher content of the elastomer;
7. SBS copolymer reduces the susceptibility of binders to altering their rheological properties occurring under the influence of short-term aging—the divergences between relaxation curves of unaged samples and those subjected to RTFOT aging simulation decrease with a higher content of the elastomer;
8. Phenomenon of asphalt binders relaxation at low temperatures can be described mathematically using a generalized Maxwell model. Out of five variants of this model, the modified generalized Maxwell model with four parameters and a constant value of time exponent (*t*^0.5^) was analyzed in detail. This allowed to achieve a better fitting of relaxation curves prepared by means of empirical and analytical methods than in the case of, e.g., using a generalized Maxwell model with six parameters and a linear time scale (*t*^1^);
9. Values of parameters of the modified generalized Maxwell model used in this study (*E*_1_, *E*_2_, *η*_1_, *η*_2_ and relaxation times *τ*_1_ and *τ*_2_ calculated on their basis) are dependent on SBS copolymer content in modified asphalt binder. Furthermore, the short-term aging process (RTFOT) also has a significant effect on the values of the examined binders.

Further tests of the influence of styrene–butadiene–styrene elastomer mixed with fillers on bituminous binders ability to relaxation at low temperatures would be advised.

## Figures and Tables

**Table 1:** Values of penetration at 25 °C and softening point.

Bitumen	Penetration at 25 °C (mm/10)	Softening Point (°C)
50/70 before RTFOT	54.6 ± 0.7	50.3 ± 0.5
50/70 after RTFOT	37.1 ± 0.5	55.4 ± 0.3
3%SBS before RTFOT	63.7 ± 0.5	53.0 ± 0.7
3%SBS after RTFOT	42.2 ± 0.9	56.4 ± 0.3
4.5%SBS before RTFOT	68.5 ± 0.6	83.3 ± 0.8
4.5%SBS after RTFOT	44.4 ± 0.8	83.8 ± 4.1
6%SBS before RTFOT	72.3 ± 0.4	99.0 ± 2.4
6%SBS after RTFOT	51.1 ± 0.6	90.7 ± 1.4
7.5%SBS before RTFOT	76.9 ± 0.6	101.6 ± 0.8
7.5%SBS after RTFOT	59.9 ± 0.3	95.4 ± 2.0
9%SBS before RTFOT	83.4 ± 0.8	104.5 ± 0.7
9%SBS after RTFOT	65.7 ± 0.7	101.0 ± 0.9

**Table 2:** Values of parameters in a generalized Maxwell model with four parameters for unaged asphalt binder containing 4.5% SBS.

Model with 4 Parameters	
R^2^	0.99204
RMS Error	5909.93
SSq	2.093·10^11^
Modulus of elasticity *E*_1_, Pa	(2114 ± 6)·10^4^
Dynamic viscosity *η*_1_, Pa·s	(1612 ± 9)·10^6^
Modulus of elasticity *E*_2_, Pa	(1487 ± 4)·10^4^
Dynamic viscosity *η*_2_, Pa·s	(2317 ± 7)·10^7^

**Table 3:** Values of parameters in a generalized Maxwell model with six parameters for unaged asphalt binder containing 4.5% SBS.

Model with 6 Parameters	
R^2^	0.99966
RMS Error	1226.73
SSq	9.014·10^9^
Modulus of elasticity *E*_1_, Pa	(1534 ± 3)·10^4^
Dynamic viscosity *η*_1_, Pa·s	(2245 ± 6)·10^6^
Modulus of elasticity *E*_2_, Pa	(1268 ± 3)·10^4^
Dynamic viscosity *η*_2_, Pa·s	(252 ± 2)·10^6^
Modulus of elasticity *E*_3_, Pa	(1243 ± 2)·10^4^
Dynamic viscosity *η*_3_, Pa·s	(2794 ± 6)·10^7^

**Table 4:** Values of parameters in a generalized Maxwell model with eight parameters for unaged asphalt binder containing 4.5% SBS.

Model with 8 Parameters	
R^2^	0.99995
RMS Error	466.15
SSq	1.301·10^9^
Modulus of elasticity *E*_1_, Pa	(1357 ± 2)·10^4^
Dynamic viscosity *η*_1_, Pa·s	(2375 ± 4)·10^6^
Modulus of elasticity *E*_2_, Pa	(1180 ± 2)·10^4^
Dynamic viscosity *η*_2_, Pa·s	(2979 ± 4)·10^7^
Modulus of elasticity *E*_3_, Pa	(1105 ± 3)·10^4^
Dynamic viscosity *η*_3_, Pa·s	(378 ± 2)·10^6^
Modulus of elasticity *E*_4_, Pa	(558 ± 3)·10^4^
Dynamic viscosity *η*_4_, Pa·s	(327 ± 5)·10^5^

**Table 5:** Values of parameters in a generalized Maxwell model described with Equation (4) for unaged asphalt binder with 4.5% SBS.

Model with 5 Parameters	
R^2^	0.99988
RMS Error	725.71
SSq	3.155·10^9^
Modulus of elasticity *E*_1_, Pa	(3014 ± 6)·10^4^
Dynamic viscosity *η*_1_, Pa·s	(2666 ± 6)·10^5^
Modulus of elasticity *E*_2_, Pa	(1405 ± 4)·10^4^
Dynamic viscosity *η*_2_, Pa·s	(951 ± 6)·10^6^
*β*	(5374 ± 8)·10^−4^

**Table 6:** Values of *β* parameter.

Bitumen	*β*
50/70 before RTFOT	0.5147 ± 0.0006
50/70 after RTFOT	0.4731 ± 0.0014
3%SBS before RTFOT	0.5267 ± 0.0007
3%SBS after RTFOT	0.5038 ± 0.0008
4.5%SBS before RTFOT	0.5374 ± 0.0008
4.5%SBS after RTFOT	0.5216 ± 0.0008
6%SBS before RTFOT	0.5067 ± 0.0009
6%SBS after RTFOT	0.5089 ± 0.0008
7.5%SBS before RTFOT	0.5116 ± 0.0008
7.5%SBS after RTFOT	0.5223 ± 0.0007
9%SBS before RTFOT	0.5241 ± 0.0007
9%SBS after RTFOT	0.5175 ± 0.0007

**Table 7:** Values of parameters in a generalized Maxwell model described with Equation (5) for unaged asphalt binder containing 4.5% SBS.

Model with 4 Parameters and a Constant Value *β* = 0.5	
R^2^	0.99983
RMS Error	854.85
SSq	4.378·10^9^
Modulus of elasticity *E*_1_, Pa	(3287 ± 3)·10^4^
Dynamic viscosity *η*_1_, Pa·s	(2569 ± 6)·10^5^
Modulus of elasticity *E*_2_, Pa	(1240 ± 4)·10^4^
Dynamic viscosity *η*_2_, Pa·s	(760 ± 2)·10^6^

**Table 8:** Values of modulus of elasticity (parameters *E*_1_, *E*_2_) and dynamic viscosity (parameters *η*_1_, *η*_2_) in a modified generalized Maxwell model with four parameters.

Bitumen	*E*_1_	*η*_1_	*E*_2_	*η*_2_
50/70 before RTFOT	56.18 ± 0.04	521.64 ± 0.97	19.97 ± 0.05	1665.58 ± 4.89
50/70 after RTFOT	44.04 ± 0.06	369.80 ± 1.31	40.81 ± 0.08	3316.41 ± 7.30
3%SBS before RTFOT	38.77 ± 0.03	317.43 ± 0.66	15.01 ± 0.04	920.71 ± 1.61
3%SBS after RTFOT	48.60 ± 0.04	416.59 ± 0.88	36.68 ± 0.05	2715.84 ± 3.62
4.5%SBS before RTFOT	32.87 ± 0.03	256.87 ± 0.59	12.40 ± 0.04	759.92 ± 1.56
4.5%SBS after RTFOT	54.19 ± 0.05	458.83 ± 1.01	31.34 ± 0.06	2233.23 ± 3.75
6%SBS before RTFOT	26.15 ± 0.02	162.41 ± 0.31	12.73 ± 0.02	515.08 ± 0.25
6%SBS after RTFOT	37.39 ± 0.03	293.97 ± 0.60	18.64 ± 0.04	1211.42 ± 1.92
7.5%SBS before RTFOT	25.18 ± 0.01	155.77 ± 0.24	9.70 ± 0.02	407.80 ± 0.24
7.5%SBS after RTFOT	25.52 ± 0.02	167.14 ± 0.26	11.89 ± 0.02	558.27 ± 0.37
9%SBS before RTFOT	15.54 ± 0.01	98.33 ± 0.16	6.79 ± 0.01	262.72 ± 0.10
9%SBS after RTFOT	23.50 ± 0.01	142.01 ± 0.21	11.20 ± 0.02	467.33 ± 0.22

**Table 9:** Values of relaxation time (parameters *η*_1_, *η*_2_).

Bitumen	*τ*_1_	*τ*_2_
50/70 before RTFOT	9.28	83.41
50/70 after RTFOT	8.40	81.27
3%SBS before RTFOT	8.19	61.33
3%SBS after RTFOT	8.57	74.04
4.5%SBS before RTFOT	7.81	61.29
4.5%SBS after RTFOT	8.47	71.27
6%SBS before RTFOT	6.21	40.46
6%SBS after RTFOT	7.86	64.99
7.5%SBS before RTFOT	6.18	42.05
7.5%SBS after RTFOT	6.55	46.94
9%SBS before RTFOT	6.33	38.68
9%SBS after RTFOT	6.04	41.71

## Data Availability

Data sharing is not applicable to this article.
